# The role of the cytoskeleton at the immunological synapse

**DOI:** 10.1111/imr.12117

**Published:** 2013-10-10

**Authors:** Alex T Ritter, Karen L Angus, Gillian M Griffiths

**Affiliations:** 1 Cambridge Institute for Medical Research, University of Cambridge Biomedical CampusCambridge, UK

**Keywords:** cytoskeleton, T lymphocytes, synapse

## Abstract

It has been over 30 years since the reorganization of both the microtubule network and a ‘peculiar actin polarization’ was reported at the contact area of cytotoxic T lymphocytes interacting with target cells. Since that time, hundreds of studies have been published in an effort to elucidate the structure and function of the microtubule network and the actin cytoskeleton in T-cell activation, migration, and effector function at the interface between a T cell and its cognate antigen-presenting cell or target cell. This interface has become known as the immunological synapse, and this review examines some of the roles played by the cytoskeleton at the synapse.

This article is part of a series of reviews covering The Cytoskeleton appearing in Volume 256 of *Immunological Reviews*.

## Introduction

**Fig 1:**
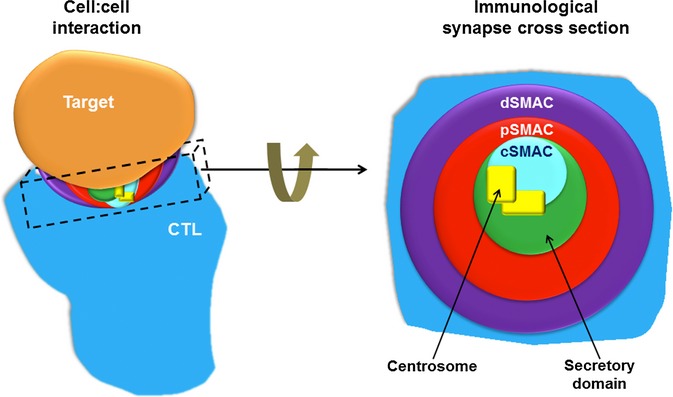
Actin and microtubule networks are polarized at the immunological synapse. The immunological synapse consists of a series of supramolecular activation clusters (SMACs) forming a bull's-eye–like ring when observed in cross section. The central SMAC (cSMAC) consists of the T-cell receptor (TCR) and associated kinases, the peripheral SMAC (pSMAC) of integrins, and the distal (dSMAC) of excluded phosphatases, such as CD45, as well as actin and actin-interacting proteins, e.g. IQGAP-1. The centrosome docks by the cSMAC and determines the point of secretory granule exocytosis in cytotoxic T lymphocytes (CTLs).

The unusual reorganization of both the microtubule network and actin cytoskeleton was noted 30 years ago 1–3. Both actin and microtubule networks take up characteristic localizations both in migratory T cells and when the immunological synapse, referred to throughout this review as ‘synapse’, forms. In migrating T cells, actin accumulates at the leading edge, as in other migratory cell types. However, T cells show a distinctive positioning of the centrosome [which is the microtubule organizing center (MTOC) in T cells], which is localized in the uropod 4 in migrating T cells, in contrast to other cell types where the centrosome polarizes toward the leading edge 5,6. The position of the centrosome changes dramatically upon target cell recognition, with the centrosome translocating from the rear of the cell all the way to the leading edge where the synapse forms 7. A mature synapse is characterized by centrosome contact with the plasma membrane at the center of the bull's-eye behind the central supramolecular activation cluster (cSMAC), with actin accumulating around the edge of the T-cell/target interface in the distal SMAC (dSMAC) (*Fig. *1). The same organization is found not only in the synapses formed between cytotoxic T lymphocytes (CTLs) and target cells but also in other cytolytic cells, including natural killer (NK) and invariant NKT cells 8, as well as in CD4^+^ T cells where the centrosome also docks within the center of the synapse 9 and actin accumulates toward the edge of the cell.

## Actin in T-cell activation

Actin dynamics and T-cell signaling are very closely linked. Not only does T-cell receptor (TCR) ligation initiate a robust actin polymerization response, but actin dynamics are also required for effective TCR signaling as inhibitors of actin polymerization disrupt T-cell activation 10–12. This is thought to be due to the fact that actin plays a role in the formation of TCR signaling clusters. TCR signaling initiates in numerous microclusters at the periphery of the synapse 13–15, which migrate toward the center where they coalesce to form the cSMAC. Actin retrograde flow has been shown to promote the centripetal movement of TCR microclusters to the cSMAC, as treatment of T cells with latrunculin, an inhibitor of actin polymerization, halts transport of TCR microclusters to the cSMAC and abrogates formation of new signaling assemblies 15,16. However, the mechanism required for this actin retrograde flow is not well understood. The actin nucleation promotion factors Wiskott–Aldrich syndrome protein (WASp), WASp family verprolin homologous protein 2, and HS1 are thought to cooperate with Arp2/3 to polymerize filamentous actin (F-actin) from the plasma membrane triggering centripetal inward movement toward the F-actin-poor cSMAC, where subsequent depolymerization is thought to occur 17,18. Myosin IIA has also been implicated in TCR microcluster translocation to the cSMAC and maintenance of synapse architecture, and this has been comprehensively reviewed recently 19.

Studies by a number of different groups have explored the role of myosin IIA in T-cell signaling as well as synapse organization and maintenance. Jacobelli *et al*. 20 demonstrated that myosin IIA accumulates at the synapse, but provided evidence that it is not required for TCR recruitment to the cSMAC. The authors conclude that myosin IIA is critical for T-cell motility, but entirely dispensable in the generation and maintenance of synapse architecture 20. Perplexingly, in 2009 a separate study presented data that completely contradicted the previous work by Jacobelli. Ilani *et al*. 21 showed that inhibition of myosin IIA activity with blebbistatin completely halts TCR microcluster movement and also that both blebbistatin and myosin IIA short-interfering RNA (siRNA) knockdown abrogates TCR signaling, measured by imaging calcium flux and staining with antibodies against phosphorylated versions of signaling proteins downstream of the TCR 21.

Recent studies exploring the role of myosin IIA in T-cell activation have not entirely ameliorated the conflict. Studies using total internal reflection fluorescence microscopy to track movement of individual SH2 domain-containing protein of 76 kDa (Slp76) or TCR microclusters after myosin IIA inhibition in T cells activated on either anti-CD3ε-coated coverslips 22 or either anti-CD3ε- 21,23 or pMHC-loaded 24,25 bilayers present a variety of results, from complete inhibition 21 or retarded microcluster movement and increased meandering 23,24 to no effect on TCR microcluster movement at all 25. Yu *et al*. 26 report that both blebbistatin (a myosin II inhibitor) and ML7 (a myosin heavy-chain inhibitor) slow the initial phase of TCR movement, whereas later phases remain unaffected. The diversity of results from these studies most likely reflects the variety of experimental systems used by different groups to test the function of myosin IIA in T cells. Differences in cell type (Jurkat versus primary T cells), T-cell activation molecule (anti-CD3ε antibody or pMHC), or substrate (coverslip versus supported lipid bilayer), as well as variation in the method of myosin IIA inhibition (pharmacological inhibition versus siRNA-mediated protein depletion) could all contribute to disparities between different studies.

Two studies show that myosin IIA inhibition reduces phosphorylation of CasL, a protein involved in mechanosensation 24,27. These studies implicate myosin IIA in the generation of force at the synapse, which may be detected through integrins. If myosin IIA activity does generate force at the synapse, the capacity of myosin IIA to affect signaling through integrins may be linked to substrate mobility. It has become apparent that stimulation of T cells using glass- or lipid bilayer-bound antigens and adhesion molecules can produce vastly different outcomes with respect to synapse formation and T-cell signaling due to differences in ligand mobility between these two systems. Ligand mobility can also be heterogeneous among different lipid bilayer systems, as variations in lipid composition can affect mobility of molecules within a lipid bilayer 28. To compensate for differences in activation parameters between the various studies examining the role of myosin IIA in T cells, it may be advantageous to adopt a live T-cell/antigen-presenting cell (APC) system. This more physiological method of T-cell stimulation may permit a more accurate assessment of how myosin IIA may cooperate with actin flow to organize the synapse, regulate signaling through integrins, or affect calcium flux into the cell.

## The immunological synapse and migratory T cells

Parallels have been drawn between the actin dynamics and molecular makeup between the leading edge of a migratory cell and the two actin-rich outer segments of the synapse, leading to the proposal that the organization of the synapse is essentially that of a radially symmetric migratory cell 29. The dSMAC, like the lamellipodium (LP), is characterized by protrusive actin polymerization that pushes out the T-cell membrane at the edge of the T-cell/target contact site 18,30,31. The lamellum (LM) is the region of a migratory cell most associated with the formation of new focal adhesions, and like the peripheral SMAC (pSMAC) is enriched in the αLβ2 integrin (leukocyte function-associated antigen-1) with integrin-associated talin used as a marker of both pSMAC and LM 31–33. Recent studies support the analogy of structures at the synapse with those found at the leading edge. Work by Yi *et al*. verifies the identity of the dSMAC as the LP and the pSMAC as the LM by staining endogenous proteins in fixed Jurkat T cells interacting with activating supported bilayers and showing that endogenous Arp2/3 and myosin IIA decorate the dSMAC and pSMAC, respectively, as they do in the LP and LM 23,32. This corroborates previous work showing cofilin and Arp3 enrichment in the dSMAC/LP and tropomyosin recruitment to the pSMAC/LM 33,34. Complementary work on anti-TCR–coated glass also demonstrates myosin IIA recruitment to the pSMAC, with some overlap in the dSMAC 22. Importantly, Yi *et al*. 23 show that myosin IIA in the pSMAC decorates concentric, bundled actin arc structures that lie just inside the dSMAC. These actomyosin contractile arcs are reminiscent of structures previously described in the LM of migrating neuronal growth cones and kidney epithelial cells 35,36. The observation that *bona fide* actomyosin contractile arc-like structures are present in the pSMAC of the synapse drives home the structural similarities between actin organization at the leading edge and at the synapse, and invites the possibility that the large body of work describing the structure and dynamics of actin at the leading edge might be used to inform T-cell biology.

Recent studies clarifying the relationship between the LP and the LM shed light on how actin in the dSMAC and pSMAC may cooperate to organize the synapse. Burnette *et al*. 35 have convincingly shown that actin arc structures in the LM are formed from bundling of branched actin that is generated in the LP. The data suggest that myosin IIA molecules bind to branched F-actin at the peak protrusion of the lamellipodium, compressing the actin into bundles of fibers, which migrate toward the interior of the cell. The majority of myosin in the kidney epithelial cells used in the study is found on these actin arc structures. Both Babich *et al*. 22 and Yi *et al*. 23 describe abundant myosin localization just inside and slightly overlapping with the actin signal in the dSMAC. Intriguingly, Yi *et al*. 23 demonstrate that TCR microclusters migrate within the pSMAC at the same rate as the actin arc-like structures, suggesting that the arcs may facilitate centripetal translocation of signaling components at the synapse, and Babich *et al*. found that SLP-76 microcluster centralization and subsequent T-cell activation were inhibited when myosin IIA and F-actin turnover was inhibited, supporting an important role for actin flow in T-cell activation. It will be interesting to see how work on the leading edge of migratory cells and the dSMAC/pSMAC of activated T cells will complement one another, as mechanisms for actin polymerization and organization are further defined in these systems.

## Control of centrosome polarization

Although the polarization of the centrosome was first observed many years ago 1,2, it is still not entirely clear how the centrosome is directed to such a specific location within the CTL synapse, nor how the centrosome interacts with the plasma membrane. What is clear is that in T cells the centrosome polarizes in response to TCR activation 7,37–42. Several downstream signaling proteins involved in transmission of the intracellular signaling from TCR have been implicated in the control of centrosome polarization. Lck is the proximal tyrosine kinase associated with CD4^+^ or CD8^+^ T-cell coreceptors, and initial studies in Jurkat cells lacking Lck expression implicated Lck in centrosome polarization 43. However, as Jurkat cells lacking either Lck or Zap70 are able to trigger both Ca^++^ fluxes and extracellular signal-regulated kinase (Erk) activation in response to TCR cross-linking or superantigen stimulation 44, it is suggested that there are linker for activation of T cells (LAT)-independent pathways in these cells, which are not seen in primary T cells. As T cells do not develop in the absence of Lck, an inducible Lck^Off^ mouse model was needed to examine the role of Lck in centrosome polarization in primary CTLs 45. Interestingly, the centrosome is able to polarize around the nucleus toward the synapse when Lck is turned off. However, the centrosome does not reach the plasma membrane and is unable to dock in Lck^Off^ CTLs, and consequently, target cell killing is ablated 45. Fyn appears to play a role in the polarization of the centrosome toward the synapse, as in CTL deficient in both Fyn and Lck of the centrosome was unable to polarize and remained in the uropod of the T cell 45. This effect seems to be dependent on the combined loss of both Fyn and Lck, as Fyn^−/−^ CTLs 46 and NK cells 47 kill targets as effectively as wildtype cells, suggesting that the centrosome polarization is uncompromised by loss of Fyn alone. These results also reveal that Fyn cannot compensate for loss of Lck, as loss of Lck alone disrupted centrosome polarization. The role of LAT is not entirely clear, as although LAT-deficient Jurkat cells show decreased centrosome polarization 48, only a modest reduction in CTL killing is observed in LAT-deficient CTLs 49. Studies on Zap70, the upstream kinase which phosphorylates LAT, are clearer with early studies on Zap70-deficient Jurkat 48 or with use of dominant negative 43 or kinase dead forms of Zap-70 50, showing some defects in centrosome polarization. A more recent mouse model expressing an analog-sensitive mutant form of Zap70 kinase allowed Zap70 kinase activity to be rapidly and reversibly inhibited, demonstrating defective killing activity when Zap70 catalytic activity is inhibited 51.

The centrosome is very sensitive to TCR signaling, polarizing to the synapse in response to weak signals, which are insufficient to trigger the associated granule polarization 52. This demonstrates how centrosome docking at the CTL plasma membrane allows the cell to prime itself for target cell killing without actually committing, thus ensuring execution of an appropriate immune response. Intriguingly, NK cells are able to polarize both centrosome and secretory granules to the synapse in response to either inhibitory receptor signals 53 or integrin activation alone 54,55, suggesting that the signaling for these events can be separated and that the control of centrosome and granule polarization may differ in CTLs and NK cells. There also appear to be some differences in signaling between mouse and human NK cells, with activating receptor engagement required to trigger granule polarization in mouse NK cells 56 but not in human NK cells 53.

## The role of calcium signaling in control of centrosome polarization

T-cell activation upon TCR engagement involves calcium signaling, but the role of calcium signaling in centrosome polarization toward target cells has been queried recently. Most published work focuses on the role of calcium signaling in mediating granule polarization and release, with little work focusing only on centrosome movement, potentially as it can be difficult to temporally separate these events. In some of the original work by Kupfer and colleagues, the accumulation of talin at the contact site between immortalized helper T cells and APCs was unaffected, but centrosome polarization toward the target cell dramatically increased by supply of exogenous calcium 38. Many years later, experiments on Jurkat cells supported this claim, as very little centrosome polarization toward anti-CD3-coated slides was observed in the absence of extracellular calcium 48. However, the authors were unable to determine how polarization of the centrosome depended on calcium: Inhibition of either calcineurin- or calcium/calmodulin-dependent kinase had no negative impact on the ability of the centrosome to polarize 48. These studies did not assess changes in intracellular calcium levels upon TCR engagement, but recent experiments have shown that TCR stimulation of CTLs carried out in medium lacking extracellular calcium resulted in an intracellular calcium flux with a similar magnitude to that which occurred in medium with calcium present 57. Although this study assessed the extent to which granule polarization relied on calcium flux, its role in centrosome polarization control was not examined 57. This area of research has been further complicated by studies carried out on primary helper T cells binding to peptide–MHC-coated glass coverslips: both intracellular and extracellular calcium were chelated, but centrosome polarization was unaffected 58. Furthermore, the cSMAC accumulating protein kinase C-θ (PKC-θ) 59, which is activated by diacylglycerol (DAG) but not calcium, has been implicated in control of centrosome polarization 60, and NK cells from patients mutated in the plasma membrane calcium channel protein ORAI1 are able to polarize their centrosome and granules normally when conjugated with target cells in the absence of any calcium flux 61.

It is difficult to explain these contrasting findings. However, each of these studies uses different cell types, and it is possible that differences in calcium dependence for centrosome polarization may exist between NK cells and T cells, as well as between immortalized cell lines and primary cells. Furthermore, recent data have revealed that intracellular calcium released from the secretory granules themselves within CTLs controls granule secretion from primary human CTLs 62, raising the possibility that relatively small and localized fluxes in calcium might be sufficient to trigger granule and presumably centrosome polarization, which may fall below the threshold of detection in some experimental situations.

## Role of the motor protein dynein

Investigations into how the force required to move the centrosome could be generated have looked at microtubule motors. Interference with plus end–directed movement along microtubules fails to interfere with centrosome polarization 7, but studies on dynein suggest that perhaps it is the minus end–directed action of this motor protein that pulls the centrosome toward the synapse. Concomitantly, dynein accumulates at the synapse, relying on the earlier recruitment of DAG 58, as well as segregation of the SLP-76 adapter protein adhesion- and degranulation-promoting adapter protein (ADAP) into the pSMAC 63. Depletion of dynein heavy chain in Jurkat cells have been reported to cause defects in centrosome polarization toward the synapse 64, and ADAP depletion prevents both dynein accumulation and centrosome polarization to the synapse in human T cells, although not in mouse T cells 63. Because ADAP interacts with dynein and microtubules, it has been proposed that clearance of ADAP to the pSMAC could cause the microtubule tension required for dynein to pull the centrosome toward the synapse 63. This is supported by data in which microtubules are seen anchored at the pSMAC, but not the cSMAC, and the centrosome drawn to the synapse by a microtubule sliding mechanism 65. However, as ADAP may not have a role in centrosome polarization in mouse cells 63 and as a more recent study on primary mouse T cells showed that neither a dynein inhibitor nor depletion of dynein heavy chain affects centrosome polarization 66, the role of dynein was unclear. More recently, shRNA has been used to deplete dynein heavy chain (DynHC) in T cells, and while DynHC depletion or blebbistatin treatment reduce centrosome polarization modestly, the effect is more pronounced when cells are both depleted of DynHC and treated with blebbistatin, suggesting that myosin IIA and dynein might act together in centrosome polarization 67.

Another unusual aspect of centrosome movement in CTLs is the ability of the centrosome to dissociate from its tight association with the nuclear envelope. Both in the uropod in migrating cells and when the synapse is formed, the centrosome and nucleus can appear to be some distance apart 4,45. The ability to dissociate from the nucleus does not seem to be a factor in controlling centrosome polarization, as the centrosome can still polarize to the synapse efficiently and CTL cytotoxicity remains the same as in control CTLs when the centrosome is artificially tethered to the nucleus 68.

## Cytoskeletal regulators of microtubules and actin at the synapse

**Fig 2:**
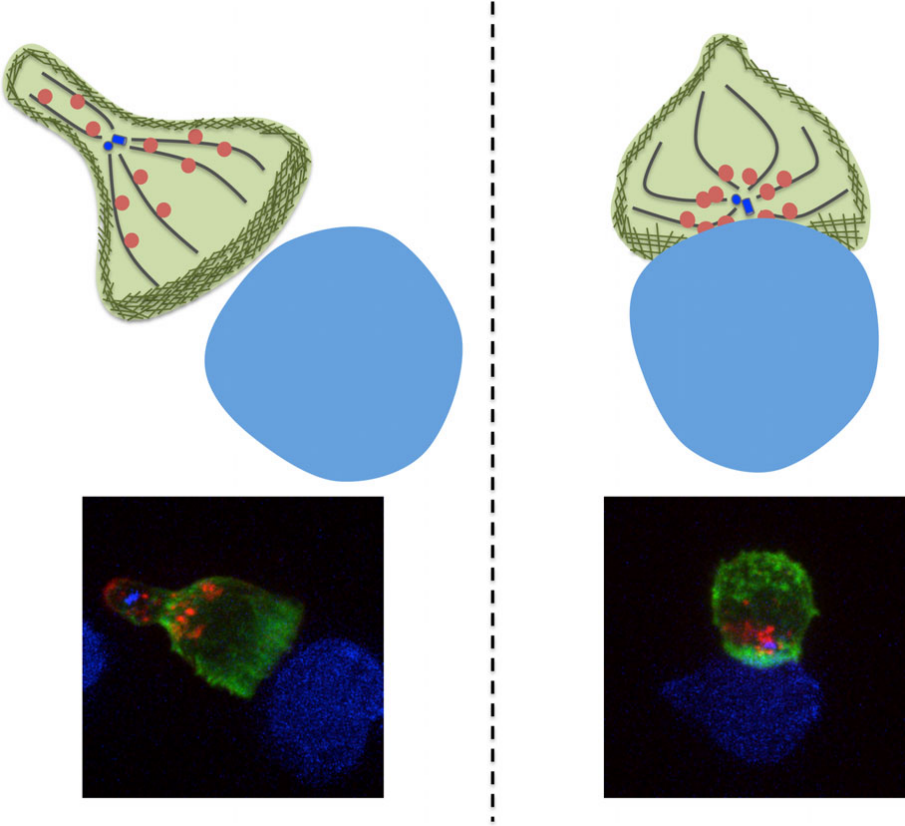
Centrosome polarization brings lytic granules to the synapse. The actin cortex of a migrating cell is continuous and intact, which provides a barrier to restrict unwarranted secretion of lytic granules that lie along microtubules. Upon target recognition, the centrosome polarizes to the synapse, bringing with it lytic granules that cluster at the centrosome. Close apposition of the centrosome with the plasma membrane brings microtubules in close proximity to the membrane at the synapse. Because actin cortical density is low at the synapse, granules that come in close contact with the plasma membrane are able to tether and fuse independent of myosin Va, releasing their cytolytic contents toward the target cell.

Actin reorganization correlates with the polarization and docking of the centrosome at the synapse (*Fig. *2), suggesting a mechanistic link between these two events 7. A number of studies have examined the role of proteins that link to microtubules or the actin cytoskeleton in centrosome polarization. The microtubule destabilizing protein stathmin 69 has a role in controlling microtubule dynamics in migrating T cells 70 and localizes to the synapse when phosphorylated 71. In stathmin knockout mice, small but statistically significant decreases in centrosome polarization and CTL-mediated killing were documented compared to wildtype cells, with a defect in PKC-θ accumulation at the synapse but normal TCR signaling 71. This is particularly interesting in the light of studies implicating PKC-θ in centrosome polarization control 60. These studies suggest that stathmin may play a small but significant role in centrosome polarization which could feasibly be linked to its capability to destabilize microtubules 69, especially as microtubule dynamics were slower in stathmin knockout compared to wildtype cells 71.

Other proteins that could affect centrosome polarization via actin have also been studied. Ezrin is an adaptor protein that can link membrane components with the actin cytoskeleton 72. Ezrin localizes with actin in the periphery of the Jurkat cell synapse in response to TCR signaling 73 and also interacts with and recruits Zap70 to the synapse 74. More recent studies find silencing of ezrin expression in T cells to impair centrosome polarization, particularly its close apposition to the stimulatory surface, with microtubules not stably attaching at the pSMAC 75. Another candidate that has been implicated in this pathway is discs large 1 (Dlg1), which has previously been linked to lymphocyte activation 76 and interaction with Lck 77. Dlg1 colocalizes in the periphery of the synapse with ezrin but not if ezrin is depleted 75. Depletion of Dlg1 itself produces similar phenotypes to those observed upon ezrin depletion 75. The authors suggest that these proteins coordinate cytoskeletal control at the synapse by ezrin providing a link between cortical actin and Dlg1, which is posited to interact with microtubules via a complex of proteins at the pSMAC 75.

IQGAP-1 can also provide a link between microtubule plus ends and the actin cytoskeleton 78, leading to the speculation that it could have a role in the mechanism of centrosome polarization to the synapse. Interestingly, although IQGAP-1 clears to the dSMAC and colocalizes with actin at the CTL synapse 7, CTLs from an IQGAP-1 knockout mouse show IQGAP-1 to be dispensable for CTL centrosome polarization and target cell killing 79, even though it is required for NK-cell centrosome polarization and cytotoxicity 80 and is claimed to be required for centrosome polarization in Jurkat cells 18. As TCR activation is shown to be impaired in the IQGAP1 knockout mouse, it is possible that compensatory mechanisms during T-cell development might mask a role for IQGAP1 in CTLs.

IQGAP-1 is a target molecule of Cdc42 and Rac1 81. Overexpression of dominant negative forms of Cdc42 prevents centrosome polarization, but actin polymerization is unaffected 82. In addition, a defect in centrosome polarization caused by shRNA-mediated depletion of Cdc42 or Rac1 in Jurkat cells can be rescued by expression of the FH2 domain of the INF2 formin, which regulates the stability of microtubules, and if depleted by minor amounts, it appears to cause a drastic reduction in centrosome polarization 83. The role of Cdc42 in control of centrosome polarization remains somewhat controversial with differing results in primary human CD4^+^ T cells 84 and Jurkat cells 18, where Cdc42 depletion does not cause defects. In fact, instead these studies suggested that it is Rac1 that plays a role in centrosome polarization 18. Interestingly, Vav1, the guanine nucleotide exchange factor (GEF) for Rho GTPases such as Cdc42 and Rac1, has been implicated in centrosome polarization control in T cells 85, but apparently its GEF activity is dispensable for its role in linking TCR signaling to actin polymerization 86, questioning the link between Vav1 and Rho GTPases in coordinated control of centrosome polarization.

Another study convincingly associates formins with centrosome polarization control: when either or both of the formins Dia1 or FMNL1 are depleted from primary human CD8^+^ T cells, the centrosome does not polarize effectively and these CTLs do not kill target cells as efficiently as control CTLs 18. FMNL1 and Dia1 localize in rings surrounding the centrosome and also with microtubules in primary T cells 18. IQGAP-1 is linked to Dia1, as it interacts with and is required for its localization in migrating fibroblasts 87. Dia1 regulates microtubules and binds microtubule tip proteins in fibroblast cells 88. If such interactions also exist in T cells, this strengthens the argument for a role of Dia1 in centrosome control in these cells.

These data strongly implicate the formin family of cytoskeletal regulators in control of centrosome polarization to the synapse; however, the coordination of the roles of Rho GTPases and IQGAP-1 in control of formins and any subsequent action on centrosome polarization requires further elucidation. As INF2 formin is implicated in microtubule stabilization in addition to a role in centrosome polarization control these two processes could potentially be linked: modest depletion of INF2 means microtubules are no longer detyrosinated, causing them to become unstable and the centrosome is unable to polarize, but both phenotypes are rescued by expression of only the FH2 domain of INF2. Impaired centrosome polarization caused by overexpression of the histone deacetylase HDAC6 is rescued by administration of a deacetylase inhibitor to CD4^+^ T cells 89, suggesting that microtubule cytoskeleton modifications are important for centrosome reorientation.

## Centrosome proteins

A large number of proteins reside at the centrosome 90, and so it seems feasible that some of these proteins may actually control centrosome polarization. Support for this concept comes from a recent study on the centrosome-localized protein casein kinase 1 δ (CK1δ) 91. CK1δ depletion from Jurkat cells abrogates centrosome polarization toward the synapse, but intriguingly, CK1δ itself is not observed at the synapse, suggesting that the protein's localization could be quite dynamic. Further investigation into the role of CK1δ found it to associate with the plus-end microtubule-binding protein EB1, and, upon CK1δ depletion, microtubule dynamics decrease 91, providing a potential link between the cytoskeleton and regulation of centrosome movement in T cells. The formin Dia1 also interacts with EB1 88, thus indicating possible involvement in the CK1δ centrosome polarization mechanism.

Paxillin also localizes at the centrosome 92,93, and is a cytoskeletal adapter protein involved at focal adhesions 94 that appears to have a role in centrosome polarization to the synapse, as a dominant negative effect is observed upon expression of only its centrosome localizing domains 95. Curiously, paxillin also localizes to the pSMAC of the synapse and so must dissociate from the centrosome to elicit its centrosome polarization function 95, as was also discovered for CK1δ 91.

The development of inducible knockout models, as used for the TCR signaling component Lck 45, will help to elucidate which proteins contribute to centrosome polarization in effector T cells. Such models will permit T cells to develop without problems that may arise due to lack of important cytoskeletal regulators.

## Involvement of the actin cortex in regulated secretion

There is much work describing the role of the actin cytoskeleton in CTL activation and signaling, but the role of the actin cytoskeleton in the cytolytic effector function of CTLs, the secretion of lytic granules, is less well understood. Due to the cytolytic nature of the secretory granule components in CTLs, precise delivery of these granules in a polarized fashion toward the target cell is important. To facilitate directed secretion of lytic granules toward a target cell, CTLs appear to utilize a mechanism of secretion that is different from secretory cells derived from the other cells which engage in regulated secretion. When a CTL encounters a target cell, it is the centrosome docking at the plasma membrane that focuses secretion at the synapse 7,96. Granules move in a dynein-mediated, minus end–directed fashion toward the centrosome, which brings them very close the plasma membrane. The role cortical actin may play in facilitating secretion in CTLs once the granules are in proximity of the PM is not clear. Actin has been shown to clear toward the periphery (dSMAC) of the synapse formed by a CTL engaging a target cell 7,97 (*Fig. *2). Although inability to clear actin at the synapse has been correlated with defective cytotoxicity 45,98, no study has directly examined the relevance of actin clearance to secretion in CTL.

The role of actin in exocytosis has been well studied in other specialized secretory cells. In cells that specialize in regulated exocytosis such as adipocytes, mast cells, pancreatic β cells, and adrenomedullary chromaffin cells, the dense network of F-actin known as the actin cortex is critical in controlling secretion. Cortical actin appears to play contradictory roles in the regulation of secretion in many cell types. One model suggests that the actin cortex acts as a physical barrier, preventing vesicles from coming close enough to the plasma membrane to dock and fuse in unstimulated cells, whereas other study models suggest that cortical actin actually facilitates trafficking of vesicles to the plasma membrane for fusion 99. The barrier model was supported by evidence that disruption of the actin cytoskeleton of pancreatic β cells with cytochalasin B induces secretion of insulin-containing granules 100. Furthermore, actin filaments lying directly beneath the plasma membrane of chromaffin cells serve to imprison secretory vesicles and prevent them from reaching the plasma membrane in the resting state, thus impeding secretion 101. Live cell imaging of stimulated secretion in these cells shows that localized actin depolymerization permits secretory vesicles to access the plasma membrane 102. Perplexingly, the same cortical actin that seems to block secretion in chromaffin cells also facilitates vesicle transport to the plasma membrane when the cells are stimulated, promoting secretion 101.

A new model that may illuminate the ‘carrier’ versus ‘barrier’ paradox of the function of cortical actin in secretion has recently been proposed with regards to exocytosis of dense-core granules in mast cells. In an exquisite study, Wollman and Meyer showed that antigen-mediated activation of mast cells results in oscillating calcium waves. These calcium waves perpetuate a periodic thickening and thinning of the actin cortex mediated by the actin nucleation promoting factor, N-WASp. The oscillation of the density of the actin cortex was shown to be critical for efficient secretion, as disruption of the actin density fluctuations or the obliteration of the actin cortex reduced the efficiency of secretion 103.

For many cells that specialize in secretion, interaction of vesicles with the actin cortex is thought to be critical for their transport to the plasma membrane. In many cases, this interaction is facilitated by myosin Va, which is known to associate with secretory vesicles in adipocytes, chromaffin cells, and pancreatic β cells 104. Secretory granules in these systems travel toward the plus ends of microtubules in a kinesin-dependent manner that brings them close to the cell periphery where they interact with the actin cortex through myosin Va. Here, fluctuations in the actin cortical density are thought to bring the vesicles close enough to the plasma membrane to mediate interaction of membrane-bound tethering factors and soluble NSF attachment protein receptor (SNARE) proteins which mediate fusion. The result is a relatively non-polarized, omnidirectional secretion of granule components. In T cells, the mode of delivery to the plasma membrane differs, as secretion needs to be focused precisely at the synapse. This is particularly important for cytolytic cells such as CTLs and NK cells, where target cell killing needs to be accurately controlled to prevent unwanted damage during an immune response. In this case, the movement of secretory granules along microtubules is reversed as granules move in a minus-end direction, thus the centrosome, the point at which microtubule minus ends are anchored in T cells, acts to define a very precise point for secretion 7.

Interestingly, myosin Va, which is important in mediating secretion in other cell types, is not required for secretion in human or mouse CTLs 105, which casts doubt on whether granule interaction with cortical actin is important in facilitating secretion in CTLs, as it is in other professional secretory cells 103,104,106,107. Thus, the current data support a model in which lytic granule movement along microtubules toward the docked centrosome brings them close enough to the plasma membrane to engage tethering and fusion-promoting factors in the two membranes to drive secretion of the granules. Interestingly, Rab27a, a protein which has been shown to interact with and recruit myosin Va to melanosomes in melanocytes 108,109, interacts with the fusion-priming factor Munc13-4, which is required for granule secretion in CTLs 110. These data point to Rab27 as a bridge between these two secretory systems: one in professional secretory cells that is dependent on plus-end microtubule transport and secretory granule interaction with the actin cortex and the other in cytolytic cells, which is dependent on minus-end microtubule transport and plasma membrane exposure to deploy secretory granules.

Two recent studies have shed light on the relationship between lytic granule exocytosis and cortical actin density in another professional cytolytic cell: the NK cell. Complementary studies using super-resolution techniques of structured illumination microscopy and stimulated emission depletion microscopy demonstrate that actin does not entirely clear from the site of NK-cell activation/degranulation 96,111. In both studies, NK cells were activated on glass coverslips coated with activating ligands or non-activating ligands, fixed after a period of time, and stained for actin and other markers. Both studies demonstrate that the density of the actin cortex at the interface of NK cells interacting with activating ligands is reduced compared to NK cells plated on inhibitory ligands and that actin is not entirely clear from the plasma membrane at the interface. A loose meshwork of actin remains, and the authors show that lytic granules preferentially localize 112 and secrete 111 at areas of actin ‘hypodensity’. Whether this pervasive actin network across the synapse remains after antigen recognition in CTLs and whether this represents a difference between CTLs and NK cells remains to be seen.

## Concluding comments

Thirty years after the unusual reorganization of both the microtubule and actin cytoskeletons was noted 1–3, much has been learned about how important these changes are for immune cell function. With the advent of new molecular and imaging technologies, the molecular events controlling the cytoskeleton and the details of the reorganization are emerging. But as with any biological system, the more we learn, the more intriguing questions are raised. It is clear that this will remain an exciting area of research in immunology with links to other biological systems for some time.
